# Supplementary material for: Chemical Compositions of Lianqiao (Forsythia suspensa) Extracts and Their Potential Health Benefits
Source: Pharmaceuticals (Basel). 2024 Jun 6;17(6):740. doi: 10.3390/ph17060740 (PMC11206439; doi:10.3390/ph17060740)
Supplement: Supplementary file 1 [file pharmaceuticals-17-00740-s001.zip › pharmaceuticals-3037220-supplementary.pdf]

Chemical compositions of Lianqiao (*Forsythia suspensa*) extracts and their potential health benefits

Boyan Gao,[a#] Hanshu Zhu,[a#] Zhihao Liu,[b,c] Xiaohua He[d], Jianghao Sun,[c] Yanfang Li,[b] Xianli Wu,[c] Pamela Pehrsson,[c] Yaqiong Zhang,[a*] Yuanhang Yao,[b*] Liangli (Lucy) Yu[b]

[a] Institute of Food and Nutraceutical Science, School of Agriculture and Biology, Shanghai Jiao Tong University, Shanghai 200240, China

[b] Department of Nutrition and Food Science, University of Maryland, College Park, Maryland 20742, United States

[c] Methods and Application of Food Composition Laboratory, Beltsville Human Nutrition Research Center, Agricultural Research Service, United States Department of Agriculture, Beltsville, Maryland 20705, United States

[d] Western Regional Research Center, Agricultural Research Service, United States Department of Agriculture, Albany, CA 94710, United States

# These authors contributed equally to this manuscript.

* To whom correspondence should be addressed:

Yaqiong Zhang, Phone: +86-21-3420-4538; Email: yqzhang2006@sjtu.edu.cn

Yuanhang Yao, Phone: +1-240-935-9440; Email: yyh2023@umd.edu

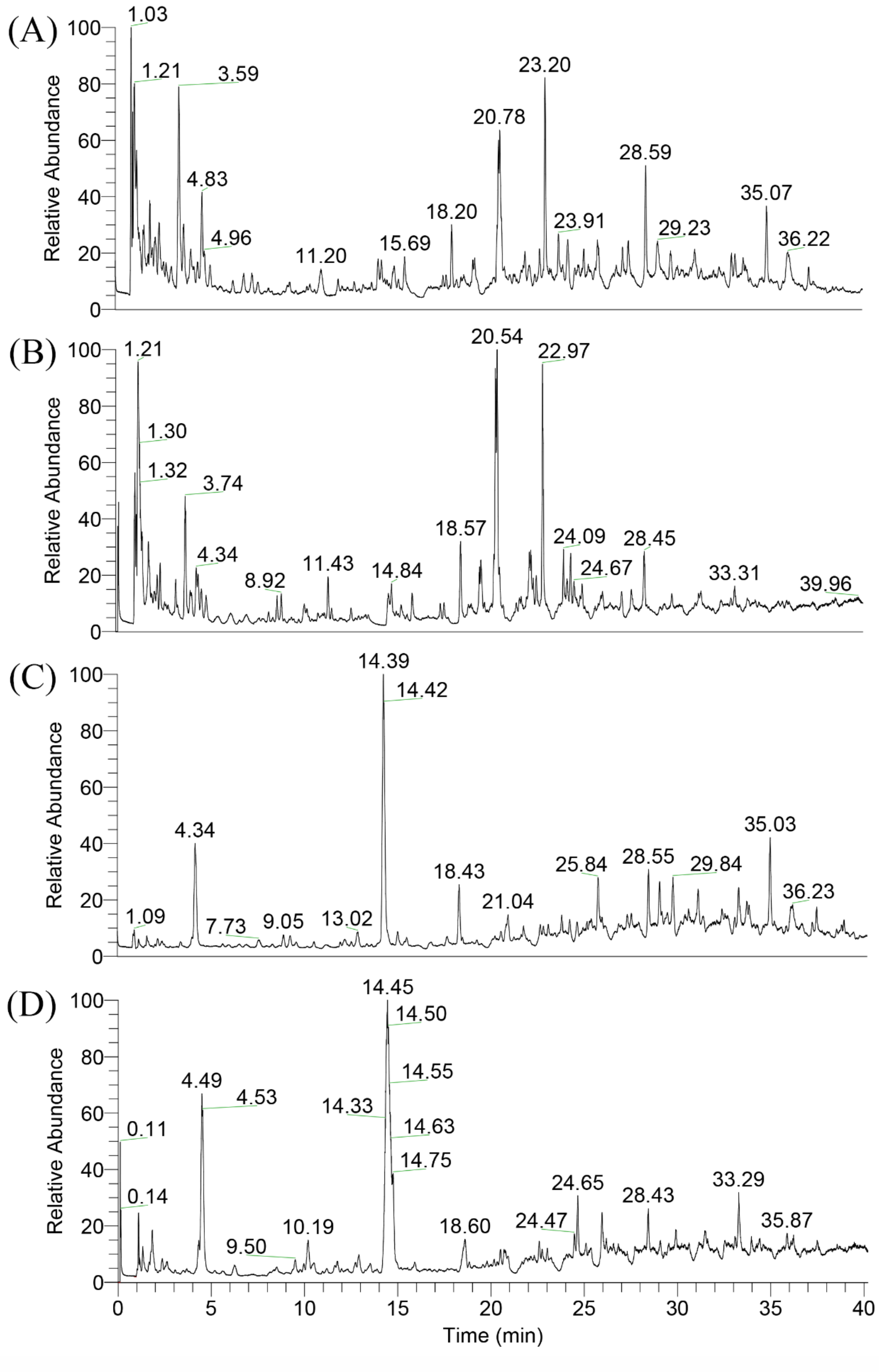

Figure S1. Chromatogram of Lianqiao water extract (WE) in positive (A) and negative (B) ionization modes, and Lianqiao ethanol extract (EE) in positive (C) and negative (D) ionization modes.
